# Genome Sequence of *Frankia* sp. Strain CH37, a Metallophore-Producing, Nitrogen-Fixing Actinobacterium Isolated from the Sea Buckthorn, Hippophae rhamnoides (Elaeagnaceae)

**DOI:** 10.1128/MRA.01184-20

**Published:** 2020-12-10

**Authors:** Jan Frieder Mohr, Anne Weiss, Sébastien Roy, Thomas Wichard

**Affiliations:** a Friedrich Schiller University Jena, Institute for Inorganic and Analytical Chemistry, Jena, Germany; b Centre SÈVE, Département de Biologie, Faculté des Sciences, Université de Sherbrooke, Sherbrooke, Quebec, Canada; Georgia Institute of Technology

## Abstract

We report the genome sequence of *Frankia* sp. strain CH37, a filamentous nitrogen-fixing soil-dwelling Gram-positive bacterium and hyperproducer of metal-complexing organic ligands (metallophores) isolated from the sea buckthorn (Hippophae rhamnoides). The 9.7-Mbp sequence, obtained using PacBio technology, harbors 7,766 predicted coding sequences, including gene clusters for metallophore production.

## Abstract (precis)

We report the genome sequence of *Frankia* sp. strain CH37, a filamentous nitrogen-fixing soil-dwelling Gram-positive bacterium and hyper-producer of metal-complexing organic ligands (metallophores) isolated from the sea buckthorn (Hippophae rhamnoides). The 9.7-Mbp sequence, obtained using PacBio technology, harbors 7,766 predicted coding sequences, including gene clusters for metallophore production.

## ANNOUNCEMENT

Frankiae are known for their ubiquity and capability to thrive as free-living soil occupants and as plant symbionts of wood angiosperms collectively termed “actinorhizal plants” (1–3). *Frankia* spp. enter host plant roots through mechanisms analogous to those found in the symbiosis between legumes and *Rhizobia* spp., resulting in the formation of root nodules which provide nitrogen to the host plant. Actinorhizal plants are environmentally significant. They are globally distributed, and their symbiosis allows them to grow under a broad range of biological conditions and stresses (4–8). The actinorhizal symbiosis plays a key role in the success of actinorhizal plants in colonizing nutrient-poor soils in the natural environment but also has great ecological, biotechnological, and economic value in applications such as land recovery, reforestation, agroforestry, and bioremediation (2).

Here, we report the genome sequence of *Frankia* sp. strain CH37, which was isolated by Prin et al. from sea buckthorn (Hippophae rhamnoides) (9), a pioneer plant used for land reclamation. Sea buckthorn has coralloid root nodules, which is the typical morphology found in actinorhizal plants (10). We decided to sequence the genome of *Frankia* sp. strain CH37 (phylogenetically classified by Ghodhbane-Gtari et al. [11]), because a previous screening of *Frankia* strains revealed that CH37 produces various metallophores in elevated amounts (12). Metallophores are a unique class of organic ligands released into the environment for multiple functions in metal management, such as metal acquisition and detoxification (12–15). Sequencing the genome of this strain was required to shed light upon metallophore biosynthetic genes and gene clusters that might be involved in metal homeostasis (16), metal detoxification of, e.g., copper (17), and plant-*Frankia* interactions (8).

*Frankia* sp. strain CH37 was provided by the culture collection of the Université Laval (Centre d'Étude de la Forêt, Québec, Canada). Bacteria were cultivated in MI medium in polycarbonate flasks for 10 days at 30°C in the dark and without shaking (12). Genomic DNA was extracted according to the cetyltrimethylammonium bromide (CTAB) protocol of the Joint Genome Institute (Berkeley, CA) (18).

Genome sequencing using a PacBio RS II sequencer, library preparation (SMRTbell template prep kit), quality control, raw read filtering, and genome assembly applying the Hierarchical Genome Assembly Process protocol (SMRT Portal v.2.2.0, RS_HGAP_Assembly.3 protocol) were carried out by a Pacific Biosciences-certified service provider (GATC Biotech AG, Germany).

Sequencing on the single-molecule real-time (SMRT) cell generated a total of 1,143,660,575 bases and 79,550 reads (*N*_50_ read length, 21,456 bp) with a mean read quality score of 0.86, resulting in 6 contigs consisting of 9,717,021 bp with a GC content of 68.9%, an *N*_50_ contig length of 9,444,267 bp, and an average reference coverage of 101×. Default parameters were used for all software packages.

The assembled *Frankia* sp. strain CH37 genome was annotated via the NCBI Prokaryotic Genome Annotation Pipeline using the best-placed reference protein set (GeneMarkS-2+) and Annotation Software v.4.13. It resulted in 7,766 predicted coding sequences, with 48 tRNAs, 3 5S rRNAs, 3 16S rRNAs, and 3 23S rRNAs. AntiSMASH analysis v.5.0 (19) revealed several secondary metabolic biosynthetic gene clusters, including those for metallophore production. The finding is consistent with previous reports about *Frankia* genome sequences (20).

### Data availability.

This whole-genome shotgun sequencing project has been deposited at DDBJ/ENA/GenBank under accession no. JADBID000000000 and consists of accession no. JADBID010000001 through JADBID010000006. The version described in this paper is the first version, JADBID010000000. It belongs to BioProject accession no. PRJNA666457 (NCBI). The SRA accession no. is SRR13065433.
